# Comparison between Nasal and Nasopharyngeal Swabs for SARS-CoV-2 Rapid Antigen Detection in an Asymptomatic Population, and Direct Confirmation by RT-PCR from the Residual Buffer

**DOI:** 10.1128/spectrum.02455-21

**Published:** 2022-02-16

**Authors:** Glenn Patriquin, Jason J. LeBlanc, Catherine Williams, Todd F. Hatchette, John Ross, Lisa Barrett, Ross Davidson

**Affiliations:** a Division of Microbiology, Department of Pathology and Laboratory Medicine, Nova Scotia Health, Halifax, Nova Scotia, Canada; b Department of Pathology, Dalhousie University, Halifax, Nova Scotia, Canada; c Department of Medicine (Infectious Diseases), Dalhousie University, Halifax, Nova Scotia, Canada; d Department of Microbiology and Immunology, Dalhousie University, Halifax, Nova Scotia, Canada; e Department of Family Medicine, Dalhousie University, Halifax, Nova Scotia, Canada; f Praxes Medical Group, Halifax, Nova Scotia, Canada; Children's Hospital Los Angeles, University of Southern California

**Keywords:** COVID-19, SARS-CoV-2, rapid, antigen, nasopharyngeal, nasal

## Abstract

Containment measures employed during the COVID-19 pandemic included prompt recognition of cases, isolation, and contact tracing. Bilateral nasal (NA) swabs applied to a commercial antigen-based rapid diagnostic test (Ag-RDT) offer a simpler and more comfortable alternative to nasopharyngeal (NP) collection; however, little is known about the sensitivity of this method in an asymptomatic population. Participants in community-based asymptomatic testing sites were screened for SARS-CoV-2 using an Ag-RDT with NP sampling. Positive individuals returned for confirmatory molecular testing and consented to repeating the Ag-RDT using a bilateral NA swab for comparison. Residual test buffer (RTB) from Ag-RDTs was subjected to real-time reverse transcription-PCR (RT-PCR). Of 123,617 asymptomatic individuals, 197 NP Ag-RDT-positive participants were included, with 175 confirmed positive by RT-PCR. Of these cases, 154 were identified from the NA swab collection with Ag-RDT, with a sensitivity of 88.0% compared to the NP swab collection. Stratifying results by RT-PCR cycle threshold demonstrated that sensitivity of the nasal collection method varied based on the cycle threshold (*C_T_*) value of the paired RT-PCR sample. RT-PCR testing on the RTB from the Ag-RDT using NP and NA swab collections resulted in 100.0% and 98.7% sensitivity, respectively. NA swabs provide an adequate alternative to NP swab collection for use with Ag-RDT, with the recognition that the test is most sensitive in specimens with high viral loads. With the high sensitivity of RT-PCR testing on RTB from Ag-RDT, a more streamlined approach to confirmatory testing is possible without recollection or use of paired collections strategies.

**IMPORTANCE** Nasal swabbing for SARS-CoV-2 (COVID-19) comes with many benefits but is slightly less sensitive than traditional nasopharyngeal swabbing; however, confirmatory lab-based testing could be performed directly from the residual buffer from either sample type.

## INTRODUCTION

To support public health efforts to reduce transmission of SARS-CoV-2 during the ongoing COVID-19 pandemic, rapid and accurate identification of the virus is paramount. While nucleic acid amplification tests (NAATs) (e.g., real-time reverse transcription PCR [RT-PCR]) are often viewed as reference methods in SARS-CoV-2 detection (1, 2), several rapid diagnostic tests (RDTs) with point-of-care (POC) applications have been authorized in Canada (https://www.canada.ca/en/health-canada/services/drugs-health-products/covid19-industry/medical-devices/authorized/list.html) and other jurisdictions (https://www.fda.gov/medical-devices/coronavirus-disease-2019-covid-19-emergency-use-authorizations-medical-devices/in-vitro-diagnostics-euas-antigen-diagnostic-tests-sars-cov-2). The most common RDT technology employed for antigen-based RDTs (Ag-RDT) is a lateral flow assay (LFIA) (2), such as the Abbott Panbio COVID-19 Ag rapid test device (3). Until recently, the Panbio Ag-RDT was only authorized for use with nasopharyngeal (NP) swabs collected from symptomatic individuals, but the assay is now available and approved by Health Canada for use with nasal (NA) swabs in symptomatic patients.

Most performance data on the Panbio Ag-RDT comes from studies using NP swabs on symptomatic individuals, often in health care settings (4–8). Performance of Ag-RDTs can vary based on a number of factors, including the specimen type, timing of collection, and the setting in which they are used (2, 9, 10). For example, compared to RT-PCR, the sensitivity for SARS-CoV-2 detection using NP swab collection with the Panbio Ag-RDTs was estimated between 70 and 80% in symptomatic individuals but increased (85% to 95%) in periods of high viral shedding (i.e., early in disease) (4–6, 11). The NP swab-containing Panbio assay has been an important component of Nova Scotia’s community testing strategies since November 2020 (https://www.canada.ca/en/public-health/services/diseases/2019-novel-coronavirus-infection/guidance-documents/use-rapid-antigen-detection-tests.html). In spite of the recent manufacturer transition from NP to NA swabs in the Panbio Ag-RDT kit, to our knowledge, there are no published data comparing the two collection methods using this kit, and Ag-RDT performance data in asymptomatic populations are limited (6, 8, 12–15). The primary goal of this evaluation was to directly compare the performance of NP and NA swab collections for use with the Panbio Ag-RDT in community-based testing of asymptomatic individuals.

Confirmatory testing at reference laboratories using molecular methods is recommended for positive, and sometimes negative, Ag-RDT results (especially in cases of high clinical suspicion), with consideration of local epidemiology (10, 16). Typically, molecular confirmation requires the collection of an additional swab. To avoid specimen recollection, a secondary objective was designed to evaluate the accuracy of RT-PCR for SARS-CoV-2 detection directly from residual buffer in specimen processing tubes used for Panbio testing. As such, our investigations not only provide data on the performance of the NA swab collection for Panbio testing but provide potential means to increase the efficiency in SARS-CoV-2 testing algorithms by expediting confirmatory testing and subsequent notifications to cases and their contacts.

## RESULTS

### Performance of Panbio test devices.

**FIG 1:**
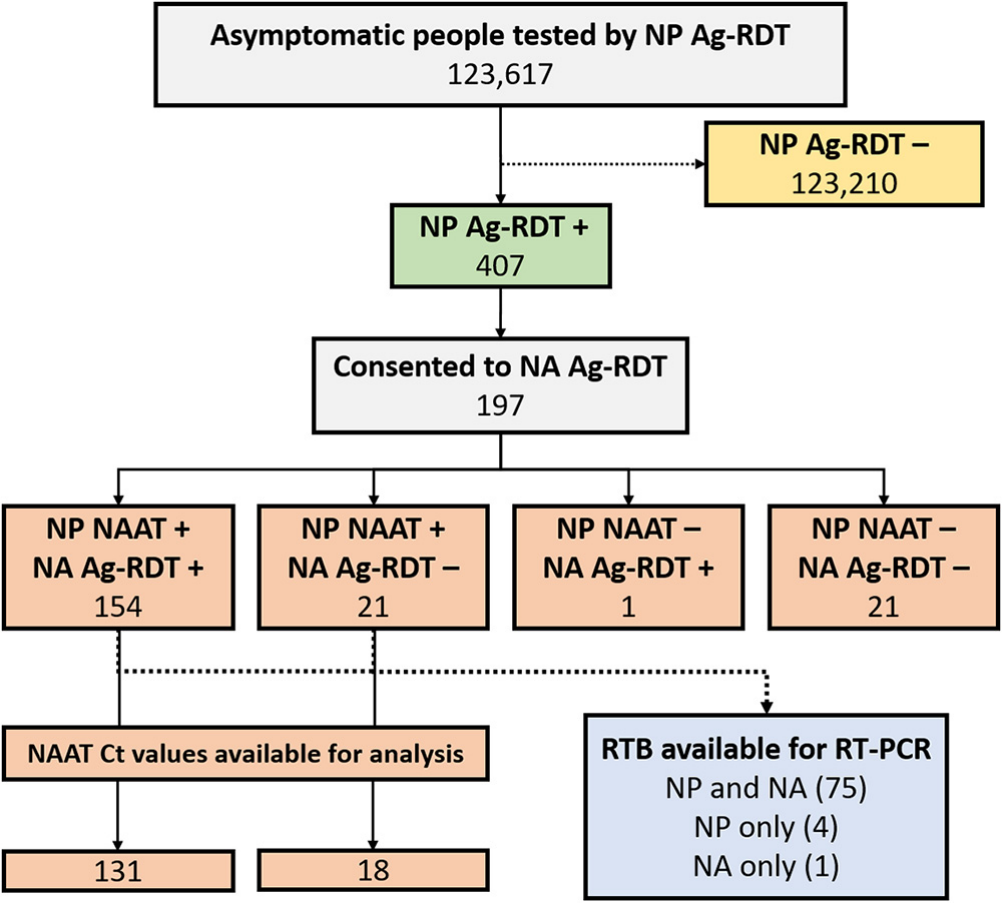
Diagram illustrating the testing outcomes of samples tested by Ag-RDT and RT-PCR from asymptomatic community members used in analysis. Ag-RDT, antigen-based rapid diagnostic test; Ct, cycle threshold; NA, nasal; NP, nasopharyngeal; RTB, residual test buffer; NAAT, nucleic acid amplification test (NAAT); RT-PCR, reverse transcription-PCR.

During the 39-day validation period in spring 2021, 123,617 asymptomatic individuals were tested with Ag-RDTs at community rapid testing sites in an urban/suburban center with a population of approximately 450,000 people. Positive NP Ag-RDTs were observed in 407 (Fig. 1). Of these, 197 people consented to have an NA swab collection, of which 175 were confirmed by NAAT as true positives. There were 21 false-positive Ag-RDT results from the NP swabs and 1 false positive that occurred in a person for both the NP and NA swabs. “Weak” test lines on Ag-RDTs from NP swab collections had a statistically significant association with false-positive results (*P* < 0.0001) and with false-negative NA test results (*P* = 0.0002) (Table S1 in the supplemental material). Given participant selection based on a positive Ag-RDT NP swab, accurate specificity calculations were not possible. On the other hand, the sensitivity of the NA swab was found to be 154/175 (88.0%; 95% confidence interval [CI], 82.2% to 92.4%) compared to the NP-based Ag-RDT.

Of the 175 positive cases confirmed by NAAT from NP swab collections in viral transport media (VTM), 149 had *C_T_* values generated from Xpert RT-PCR testing and thus were used in further analysis. The median *C_T_* value was 18.6 (range, 12.7 to 31.3). There was a significant difference in the mean *C_T_* value between true-positive NA swabs (i.e., NP positive) (18.3) and false-negative NA swabs (22.6; *P* < 0.0001). Stratification of *C_T_* values revealed a decreasing trend in NA Ag-RDT sensitivity with increasing *C_T_* values (Table 1).

**TABLE 1:** Sensitivity of the Panbio NA Ag-RDT (versus NP Ag-RDT) stratified by *C_T_* value^*a*^

*C_T_* range	Sensitivity^*b*^ (%; no. of sensitive samples/total no. of samples)	95% CI (%)
<15	100 (15/15)	78.2–100
15–19.9	94.0 (79/84)	86.7–98.0
20 to 24.9	80.5 (33/41)	65.1–91.2
25 to 29.9	57.1 (4/7)	18.4–90.1
≥30	0.0 (0/2)	0–84.2

a Ag-RDT, antigen-based rapid diagnostic test; *C_T_*, cycle threshold; CI, confidence intervals; NA, nasal; NP, nasopharyngeal; RT-PCR, reverse transcription PCR.

b Sensitivity of the Panbio NA Ag-RDT was determined from NP swabs tested by Ag-RDT, which were confirmed using real-time RT-PCR.

### Direct RT-PCR of RTB.

To investigate the possibility of using residual test buffer (RTB) from Ag-RDT testing as a confirmatory strategy, a subset of available extraction tubes from NAAT-positive patients from the asymptomatic testing site was used for RT-PCR (Fig. 1). RTB was available for both NP and NA swabs for 75 patients, for NP swabs only in 4 patients, and for NA swabs only in 1 patient. All 79 NP RTBs were positive by RT-PCR for a sensitivity of 100.0% (95% CI, 95.4 to 100.0%). For the 76 nasal swab RTBs, 72 were positive by RT-PCR, and 3 were indeterminate results, for a combined sensitivity of 98.7% (95% CI, 92.9% to 100%). Using a combined sensitivity for positive and indeterminate results in this context is justified, as public health initiates contact tracing and isolation in both cases until results are confirmed by NAAT from NP swabs in VTM. The samples included the RTB from eight false-negative NA Ag-RDTs. Of these, five had a positive RTB RT-PCR, two were indeterminate, and one was negative. Though categorical agreement between NAAT from NP in VTM and from RTB was robust, the direct correlation (*R*^2^ values) of available *C_T_* values between sample types was low (Fig. S1).

## DISCUSSION

Our data define a discrete range of *C_T_* values detected by NP and NA swabs applied to the Panbio Ag-RDT, consistent with the work of others for this testing platform using NP swabs (5, 8, 17), NA, or saliva samples (6, 18), and for other Ag-RDTs using various specimen types (19–22). Others have compared the performance of Panbio NA versus NP swabs in a mixture of symptomatic and asymptomatic individuals (6, 18, 23), but few studies have targeted asymptomatic populations directly using Panbio (13) or other Ag-RDTs (24, 25). Here, the sensitivity of bilateral NA swab sampling and testing using the Panbio Ag-RDT was 88.0% compared to confirmed SARS-CoV-2 cases initially detected by NP-based Ag-RDT. Data from the manufacturer product insert suggest the sensitivity of the Panbio Ag-RDT NA swab collection is 98.1% compared to RT-PCR performed on NA swabs and 91.1% compared to NP swabs for RT-PCR. However, there are no data directly comparing the performance of NA versus NP swab collections for use with Panbio Ag-RDT. Although there is reduced sensitivity compared to using NP swabs in our evaluation, the NA swab offers several advantages. It is less invasive, which is more acceptable by people compared to NP swabs (26, 27), and the ease of use facilitates volunteer training and self-testing at home or workplace (28, 29). These factors, combined with rapid turnaround time, low cost, and ease of use afforded by Ag-RDTs (30–32), can help facilitate repeat testing over time and may compensate for reduced sensitivity (5, 9, 33).

Not surprisingly, the *C_T_* values associated with false-negative NA swabs were significantly higher than those associated with true-positive NA swabs, suggesting that patients with a lower viral load would be more likely to be missed by this collection specimen type. This is further demonstrated in stratified *C_T_* values, where sensitivity progressively declined in higher *C_T_* categories. Importantly, *C_T_* values less than 20 were reliably detected by NA sampling, and those above 20 may lead to a substantial proportion of false negatives; however, some investigations have demonstrated a link between Ag-RDT positivity and viral viability or infectiousness (34–38), especially for NP collection (39), and therefore, hypothetically, missed cases by Ag-RDT may be less impactful on transmission, though more data are needed to draw conclusions in this regard. Although this project was not aimed at determining the sensitivity of the NP sample as applied to the Ag-RDT (given only positive results triggered NA swab collection), the proportion of positives with *C_T_* values greater than 20 suggests that the NP sample collection is substantially more sensitive than NA sampling. This suggests that NP sampling for Ag-RDT may identify cases earlier in their infection than NA sampling and therefore may be favored in situations of high prevalence, when earlier detection may reduce community transmission. Though we, and others (40), comment on the association of a “weak” band and false-positive NP swab Ag-RDTs, it is important to note that the manufacturer’s instructions make no distinction between band intensity, and manufacturer’s instructions should be strictly followed to avoid jeopardizing the validity of results.

**FIG 2:**
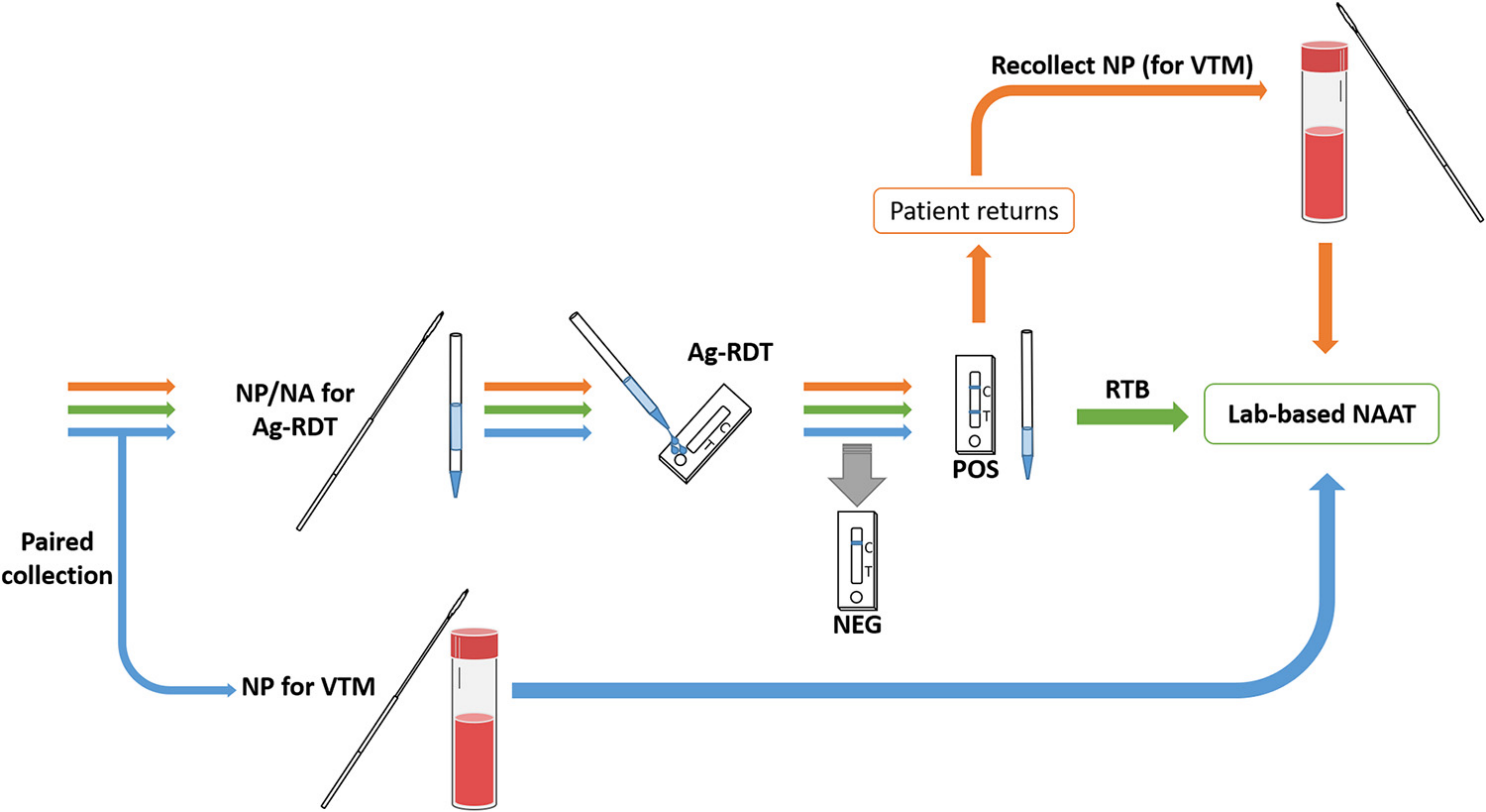
Possible confirmation strategies for Ag-RDT results using a laboratory-based NAAT. Typically, confirmation of Ag-RDT results relies on (i) the recollection of an additional specimen (e.g., NP in VTM) for NAAT testing following a positive Ag-RDT result (orange arrows); or (ii) performing individual paired collections for Ag-RDT and RT-PCR and comparing results of the Ag-RDT and the laboratory-based NAAT (blue arrows). A third strategy was investigated to reduce the need for paired collection, or return for recollection, where NAAT testing is performed directly on RTB following a positive Ag-RDT (green arrows). Ag-RDT, antigen-based rapid diagnostic test; NA, nasal; NP, nasopharyngeal; NAAT, nucleic acid amplification test; RTB, residual test buffer; VTM, viral transport media.

Current guidelines advise confirmation of positive Ag-RDTs with a sensitive and specific molecular method, such as RT-PCR (16). Recollection requires contacting the patient, arranging additional testing, and transport to the lab, all of which may result in prolonging the interval before contact tracing may commence. While paired concurrent collection of two NP swabs for parallel testing by Ag-RDT and RT-PCR could alleviate this problem, there would likely be reduced patient acceptability of two sequential NP swabs, and testing efficiency might suffer given the extra time commitment required for these two collections. The ability to test residual Panbio test buffer (RTB) from positive Ag-RDT samples would greatly facilitate this process. These results support other work (41) and show that positive Ag-RDTs can be confirmed by direct RT-PCR on RTB with great reliability (Fig. 2), thereby reducing the proportion of those needing to return for subsequent NP swab for VTM. This benefit, however, may be countered by other case-finding/notification opportunities that arise when one returns for confirmatory testing (such as counseling, education, and inviting household and close contacts for targeted testing).

There are several limitations to this study. Molecular testing was not performed on all individuals presenting to the community-based testing sites; therefore, we cannot comment on the true sensitivity of the NP swab and Panbio Ag-RDT compared to a molecular reference. However, our findings provide a reliable comparison between the performance of the NP and NA swabs using the Panbio Ag-RDT. As these were performed at community testing sites, which, by design, collect minimal personal and clinical information, screening criteria were used to define our population as being asymptomatic and having no known exposures to SARS-CoV-2 cases. This method would not account for incomplete or inaccurate self-reporting from participants or individual variations in subjective self-assessment of symptoms. In addition, some positive Ag-RDT tests led to targeted testing of household members and close contacts for whom the screening criteria would not have necessarily applied. Though we and others (23) demonstrate positive RTB RT-PCR in the setting of false-negative Ag-RDTs, further work would be required to determine if direct RT-PCR on RTB could be used to reliably rule out SARS-CoV-2 in those with negative Ag-RDTs. In this way, confirmation from RTB may be thought of as a way to rule in, but not rule out, detection until further validation is performed. In our method, the addition of phosphate-buffered saline (PBS) required for adequate sample volume may also inadvertently lead to failures of assays that use endogenous human gene targets as a control. Of note is the importance of validating any sample medium/matrix used for molecular testing platforms to ensure compatibility. Finally, our investigation occurred during a period with an average of 9.5 new positive RT-PCR cases per 100,000 (range of 1.0 to 23.3) per day, so positive predictive value of the Ag-RDT would differ in locations of lower or higher prevalence.

As multiple testing modalities for SARS-CoV-2 are being granted emergency use authorization and are being used to fulfill specific off-label needs, local clinical validation is critical to understanding the deficits that could detract from the benefits of Ag-RDTs. Once the performance of a test can be established for a particular population and an implementation plan is determined (42), one can apply the procedure and results with confidence, recognizing and adapting to the recognized limitations (9). Ultimately, in our case, the ease of use and rapid results of these assays has have allowed for the testing of thousands of people who otherwise would not have been tested. Despite the reduced sensitivity compared to RT-PCR, Ag-RDTs in this expanded testing capacity have been able to detect cases in asymptomatic people, allowing for the early initiation of public health measures to reduce transmission. The added acceptability of nasal swabs will hopefully help people in the community continue to make regular testing an ongoing part of COVID-19 mitigation strategies.

## MATERIALS AND METHODS

### Inclusion criteria.

All samples were collected from 23 April to 31 May 2021 from attendees of community-based rapid COVID-19 testing sites as part of a community engagement and testing strategy in Nova Scotia, Canada (https://www.canada.ca/en/public-health/services/diseases/2019-novel-coronavirus-infection/guidance-documents/use-rapid-antigen-detection-tests.html). In order to be tested, all attendees were screened upon arrival to ensure they were asymptomatic, had no close contact with known or suspected cases, and had not been at a public location identified by Public Health as having increased risk of transmission. Those who screened positive (due to symptoms, having been exposed to a known positive case, or having been present at an identified location with increased transmission) were not included in this validation population but, instead, were referred to a primary assessment center for RT-PCR testing.

### Specimen collection.

NP swabs were collected from all attendees using the flocked swab included in the Panbio test kit. Those performing specimen collection were health care providers or community-based volunteers trained with a standard technique to ensure consistent sample quality, using the “4Ds” NP swab collection model, direction, depth, duration, and dialing (https://vimeo.com/516853275/c67017fd3a). If the Panbio test results were positive for the NP swab, individuals were contacted, and verbal consent was obtained to collect a bilateral nares swab prior to the NP swab required for confirmatory testing using molecular methods. This work was a quality initiative.

### Antigen testing.

Testing was performed onsite on NP swabs using the Abbott Panbio COVID-19 Ag rapid test device (Abbott Rapid Diagnostics GmbH, Orlaweg, Germany) according to the manufacturer’s instructions. Negative results were reported to the individuals using a secure text messaging system. Those with a positive Ag-RDT were asked to return for recollection of an NP swab for a confirmatory molecular test. For those who consented, a bilateral NA swab was collected in addition for use in the validation. NA swabs were processed for Ag-RDT in the same manner as NP swabs. Recollections occurred within 3 h of the initial positive antigen test. Test lines were considered “weak” or “strong” at the subjective discretion of the testing site lead.

### Molecular confirmation of Panbio-positive results.

NP swabs for confirmatory molecular testing were collected by a health care professional, placed in viral transport medium (VTM) (virus sampling kit; Yocon Biology Technology Company, Beijing, China), and sent to the reference laboratory. Molecular confirmation was carried out according to the manufacturer’s instructions using Xpert Xpress SARS-CoV-2 or Xpert Xpress SARS-CoV-2/Flu/RSV assays (Cepheid, Sunnyvale, CA), the Aptima SARS-CoV-2 assay on the Panther system (Hologic, Inc., San Diego, USA), or the cobas SARS-CoV-2 test on the 6800 instrument (Roche Diagnostics, Meinheim, Germany) (43). Where available, cycle threshold (*C_T_*) values were recorded. For consistency, only *C_T_* values derived from the Xpert SARS-CoV-2 FluA/B/RSV assay were used for the *C_T_* stratification analyses, as this platform was used for the majority of samples. Positive and negative RT-PCR results were communicated to the patient, and any positive results were also reported to Public Health for further management and case contact tracing.

### Direct RT-PCR of residual Panbio buffer.

Residual test buffer (RTB) used in Panbio testing of NP and NA swabs, along with the NP swab in VTM, was transported to the reference laboratory and held at 4°C until processing. To provide adequate volume, 500 μL of sterile 1× phosphate-buffered saline (PBS) (Gibco, Thermo Fisher Scientific) (44) was added to the RTBs, which were subsequently tested using the Roche cobas 6800 assay according to the manufacturer’s specifications. *C_T_* values of the Orf1a target were used in the analyses, but E gene results were also collected. Specimens with a single target-positive result were considered indeterminate. Individuals with complete specimen sets (i.e., NP swab RTB, NA swab RTB, and NP swab in VTM for confirmatory RT-PCR) were included in the analyses. In all comparisons, descriptive statistics were performed with QuickCalcs (GraphPad Software, San Diego, USA; https://www.graphpad.com/quickcalcs).
