# Supplementary material for: Comparison between Nasal and Nasopharyngeal Swabs for SARS-CoV-2 Rapid Antigen Detection in an Asymptomatic Population, and Direct Confirmation by RT-PCR from the Residual Buffer
Source: Microbiol Spectr. 2022 Feb 16;10(1):e02455-21. doi: 10.1128/spectrum.02455-21 (PMC8849095; doi:10.1128/spectrum.02455-21)
Supplement: SUPPLEMENTAL FILE 1 — Supplemental material. Download SPECTRUM02455-21_Supp_1_seq12.pdf, PDF file, 0.2 MB [file spectrum02455-21_supp_1_seq12.pdf]

Table S1. Association of weak test bands on the NP Ag-RDT with false positive results, and in patients with false negative NA Ag-RDT.

NP Ag-RDT band intensity	True positive NP Ag-RDT*	False positive NP Ag-RDT*	True positive NA Ag-RDT**	False negative NA Ag-RDT**
Strong	98	0	94	4
Not reported	58	6	70	9
Weak	19	16	11	8
P value#		<0.0001		0.0002

*A true positive NP Ag-RDT represents a patient having positive NP Ag-RDT and NAAT results, whereas a false positive is a positive NP Ag-RDT with a negative NAAT. **A true positive NA Ag-RDT represents a patient having positive NA Ag-RDT and NAAT results, whereas a false negative is a positive NAAT in a patient with a negative NA Ag-RDT. Abbreviations: antigen-based rapid diagnostic test (Ag-RDT); nasal (NA), nasopharyngeal (NP); and nucleic acid amplification test (NAAT); residual test buffer (RTB). #P-value reported for combined “Strong” and “Not reported” vs. “Weak” given the unquestionable positivity of those that were strong or unremarkable.

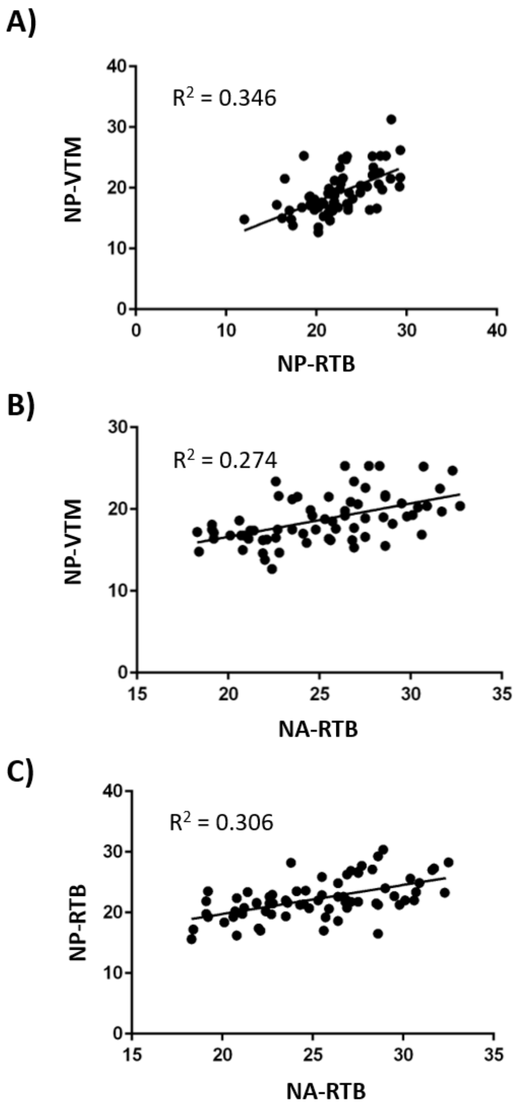

Figure S1. Correlation of Ct values between sample types in asymptomatic SARS-CoV-2-positive community dwellers. A) Ct value correlation between NP RTB and NP swabs in VTM; B) Ct value correlation between NA RTB and NP swabs in VTM; and C) Ct value correlation between NP and NA RTB. Abbreviations: cycle threshold (Ct), nasal (NA), nasopharyngeal (NP); and residual test buffer (RTB).
